# Smokey Chimneys

**Published:** 1859-12

**Authors:** 


### SMOKEY CHIMNEYS.

Next to burning wood in a fire place on old fashioned "dog irons," mineral coal in an open grate makes the most cheerful and lively fire; but as three-fourths of the heat goes up the chimney, various patterns of stoves and furnaces have been introduced as a matter of economy, because twenty dollars in money had to be expended, to get five dollars worth of warmth. An English board of health advises to make the grates broader, and not so deep, while by having a flue not over nine inches across, these advantages will be gained, a large part of the smoke will be consumed, a great deal of heat will be saved, and the tendency to smoke will be greatly diminished.

The warmer a chimney is, the better it will draw, hence the chimney should be incorporated in the wall of the building, it should be on the south side of it, and if between two buildings, it will draw better than if at an exposed end.

The flue of a chimney should be smooth on its sides, and should grow broader as it ascends, rather than narrower.

A recent improvement in coal grates in burning the smoke and gas, and saving heat, is to have the angle made by the back of the chimney, and the rear part of the flue perfect and tight, thus detaining the smoke and gas just over the fire long enough to be consumed, while any remnant left, travels three or four inches outward towards the room, and then ascends through a chink three or four inches deep just behind the arch.

